# Generative Adversarial Network (GAN) for Automatic Reconstruction of the 3D Spine Structure by Using Simulated Bi-Planar X-ray Images

**DOI:** 10.3390/diagnostics12051121

**Published:** 2022-04-30

**Authors:** Ching-Juei Yang, Cheng-Li Lin, Chien-Kuo Wang, Jing-Yao Wang, Chih-Chia Chen, Fong-Chin Su, Yin-Ju Lee, Chun-Chung Lui, Lee-Ren Yeh, Yu-Hua Dean Fang

**Affiliations:** 1 Department of Biomedical Engineering, National Cheng Kung University, Tainan 701401, Taiwan; p88041067@gs.ncku.edu.tw (C.-J.Y.); gilingking@gmail.com (J.-Y.W.); lona15926@gmail.com (C.-C.C.); fcsu@ncku.edu.tw (F.-C.S.); 2 Department of Medical Imaging, E-Da Cancer Hospital, I-Shou University, Kaohsiung City 82445, Taiwan; yinjulee@gmail.com (Y.-J.L.); ed109491@edah.org.tw (C.-C.L.); 3 Department of Orthopedic Surgery, National Cheng Kung University Hospital, College of Medicine, National Cheng Kung University, Tainan 704302, Taiwan; jengli94@gmail.com; 4 Department of Orthopedics, College of Medicine, National Cheng Kung University, Tainan 701401, Taiwan; 5 Department of Medical Imaging, National Cheng Kung University Hospital, College of Medicine, National Cheng Kung University, Tainan 701401, Taiwan; n625396@mail.hosp.ncku.edu.tw; 6 Medical Device Innovation Center, National Cheng Kung University, Tainan 704302, Taiwan; 7 Department of Radiology, E-Da Hospital, I-Shou University, Kaohsiung City 82445, Taiwan; yehlr3323@gmail.com; 8 Department of Radiology, School of Medicine, University of Alabama at Birmingham, Birmingham, AL 35294, USA

**Keywords:** 3-dimensional (3D) spine, generative adversarial network (GAN), simulated biplanar X-ray

## Abstract

In this study, we modified the previously proposed X2CT-GAN to build a 2Dto3D-GAN of the spine. This study also incorporated the radiologist’s perspective in the adjustment of input signals to prove the feasibility of the automatic production of three-dimensional (3D) structures of the spine from simulated bi-planar two-dimensional (2D) X-ray images. Data from 1012 computed tomography (CT) studies of 984 patients were retrospectively collected. We tested this model under different dataset sizes (333, 666, and 1012) with different bone signal conditions to observe the training performance. A 10-fold cross-validation and five metrics—Dice similarity coefficient (DSC) value, Jaccard similarity coefficient (JSC), overlap volume (OV), and structural similarity index (SSIM)—were applied for model evaluation. The optimal mean values for DSC, JSC, OV, SSIM_anteroposterior (AP), and SSIM_Lateral (Lat) were 0.8192, 0.6984, 0.8624, 0.9261, and 0.9242, respectively. There was a significant improvement in the training performance under empirically enhanced bone signal conditions and with increasing training dataset sizes. These results demonstrate the potential of the clinical implantation of GAN for automatic production of 3D spine images from 2D images. This prototype model can serve as a foundation in future studies applying transfer learning for the development of advanced medical diagnostic techniques.

## 1. Introduction

The human spine is an essential axial skeleton that protects the central nervous system and provides conduction to the peripheral nervous system. Clinical evaluation of the spine includes two-dimensional (2D) or three-dimensional (3D) gross structural evaluation and soft tissue analysis.

The basic physics of X-rays involves the interaction between electrons and tungsten nuclei. Computed tomography (CT), dual-energy CT, low-dose CT, quantitative computed tomography (QCT), fluoroscopy, and dual-energy X-ray absorptiometry (DXA) were developed depending on different imaging reconstruction algorithms, shapes of the X-ray beam, and energy spectra to provide diverse diagnostic information.

Traditional X-ray machines are the most popular routine imaging modality for quick one-shot 2D anatomic inspection. CT, dual-energy CT, low-dose CT, and QCT provide more spatial information, but their utility as a tool for routine orthopedic evaluation is limited owing to the machine cost and popularization rate in different levels of hospitals. The two X-ray energies have the capacity to differentiate different tissue components, and DXA is popular for bone density scans. However, DXA lacks producing high-quality images. Magnetic resonance imaging (MRI) is a 3D non-radiation-exposure system that employs hydron characteristic analysis to provide better soft tissue contrast than CT and X-ray. However, the MRI machine is expensive, and the scan timing is longer than that with other diagnostic imaging modalities.

The entire spinal column is a complex multiarticular system consisting of 24 vertebrae controlled by muscles. A single spine vertebra is a polymorphic geometric structure comprising the body, pedicle, lamina, and spinous processes with diverse shapes and angles. As a result, 2D image evaluation has a deficiency in human interpretation of the exact 3D anatomic location. Building 3D structures of the spine can provide more informative assessments for explanation of disease, diagnostic and therapeutic purposes. Currently, the more reliable and faster imaging modalities for 3D bone structural analysis are CT and 3D tomography [1], which incur more exposure to ionizing radiation doses than plain film X-ray images do [2]. A reasonable reduction in ionizing dose exposure in orthopedic patients is an important issue [3,4].

From a clinical perspective, there is no perfect diagnostic machine or algorithm. The essential issue is choosing an appropriate diagnostic tool for the desired diagnostic purpose. This study focused on developing a cost-effective method for building 3D gross anatomic structures from 2D images as an auxiliary diagnostic tool.

Approximately 40 years ago, Openshaw et al. studied thoracic bone measuring ratios on X-ray and CT examinations [5]. Related stereoradiographic research also involves 2D to 3D transformation. In 1981, Szirtes proposed contour radiography and assumed that two divergent projections from two X-ray sources could produce distinct image lines [6]. In 1988, Dansereau et al. applied a similar concept to contour radiography, using paired X-ray images to reconstruct the entire rib cage [7]. Other spinal structure analysis studies have been studied on approaching the shape using 2D image landmarks and fitting functions [8,9,10]. A subsequent study with more specific vertebral landmarks was performed to provide a reliable 3D reconstruction of the spine [11]. Two/three-dimension (2D/3D) image registration in different domains is an important research topic in the field of computer vision. The aforementioned studies were based on a linear transformation of the geometry. Additionally, Cottes et al. [12] introduced a statistical model, the point distribution model (PDM) [12], in which the predicted shape arises from a combination of the mean shape, eigenvectors (feature components), and weighting parameters. The PDM was built using a sufficient collection of datasets to span the space adequately to the predicted shape. In 2003, Benameur et al. demonstrated that statistical models performed better than conventional 3D reconstruction methods for the spine [13]. Semi-automatic methods and validation of the 3D reconstruction of spines from bi-planar images have been developed [14,15,16,17]. The EOS imaging device was commercially available in 2007 and enabled simultaneous reconstruction of 3D spine segments from self-calibrated biplanar radiographs [18,19]. The EOS system is a low-dose and quick examination modality for approaching 3D structures; however, this semi-automatic system relies on bony landmark labeling by medical experts. In 2021, Bennani et al. used uncalibrated biplanar radiographs with bounding boxes and an active shape model (ASM) to reconstruct 3D vertebrae [20]. These methods involve two important components: (1) feature extraction or bone landmark labeling and (2) building 2D/3D image registration models. These methods are semi-automatic, and sufficiently precise bony landmark labeling manually by a medical expert is required. Such a process is time-consuming, laborious, and depends on observer labeling reliability [21,22] to avoid error prediction in 2D/3D image registration models. Moreover, updating the statistical models for specific patient groups is expensive [23].

In 2014, the idea of a generative adversarial network (GAN), invented by Goodfellow [24], became one of the most important and interesting architectures for machine learning (ML). Compared to traditional linear transformation models, statistical models, and convolutional neural networks (CNNs), GAN is a probabilistic generative model that contains two types of neural networks: a generator and a discriminator. GAN is an automatic optimization solution method that is capable of feature extraction and building the predicted model simultaneously by the trade-off between the generator and discriminator with creative characteristics. For example, GANs are often applied to images to solve the problem of transforming low-resolution images into high-resolution images from other datasets [25,26], image-to-image translation [27], present depth information in 2D images [28], and produce 3D models from a probabilistic latent space [29]. The basic prototype of the GAN uses random signals as inputs to the generator network and transforms these random signals into meaningful fake outputs. The discriminator network further judges fake and ground truth (GT) objects. Because the GAN architecture involves training two neural networks, its technical implantation is challenging.

The conditional GAN was proposed by Mirza and Osindero [30], who introduced conditional constraint inputs to the basic GAN model for specific mapping modeling purposes. In 2017, Isola et al. proposed pix2pix-GAN [31] using L1+ cGAN loss and Patch-GAN for discriminators that produce high-quality image-to-image translation tasks. Based on these studies, Ying et al. proposed an X2CT-GAN [32] that can transfer biplanar chest X-ray images to a 3D CT volume. These studies support our hypothesis of reconstructing 3D spines from bi-planar orthogonal 2D images, and we consider GAN as a potential ML method for automatic transfer of bi-planar 2D/3D image registration of the spine.

In this study, we modified the X2CT-GAN with ResNet [33] as the backbone for feature extraction and incorporated the empirical experience of a radiologist to tune the input signals to accomplish an end-to-end method for the automatic transfer of simulated bi-planar X-ray images for 3D spine structures. Furthermore, two different signal condition inputs—the original bone signal and enhanced bone signal conditions—with different dataset sizes (333, 666, and 1012) were used to compare the 3D reconstruction performance. This study demonstrates the potential clinical application of GAN for 2D/3D image registration of the spine and provides implantation details in engineering and clinical considerations to build a prototype for transfer learning of similar tasks in the future.

## 2. Materials and Methods

### 2.1. Data Collection

We retrospectively collected chest CT data from E-Da Hospital, National Cheng Kung University (NCKU) Hospital in Taiwan, and The Cancer Imaging Archive (TCIA) [34,35] under institutional review board certification. We applied the following exclusion criteria to exclude the abnormal spinal structure for constructing a basic GAN model: (1) post-metallic implants, (2) post-vertebroplasty, (3) post-laminectomy, (4) post-discectomy, (5) tumor invasion, (6) severe scoliosis, and (7) fracture destruction.

The data included 1012 non-contrast-enhanced chest CT images of 984 patients (217 men, 159 women, 608 unknown genders; available age, 49.88 years ± 10.70 (mean ± SD)) (Table 1).

The CT data contained 0.6–10 mm (3.47 mm ± 1.76 (mean ± SD)) slice thickness, 200–500 mm (347.01 mm ± 37.05 (mean ± SD)) reconstruction diameter/field of view (FOV), 100–140 kVp (123.06 kVp ± 6.33 (mean ± SD)) tube voltage, 38–1080 mA (199.78 mA ± 185.64 (mean ± SD)) tube current, and 512 × 512 pixels for each 2D image (Table 2).

The GT of the spine was labeled using an in-house semi-automatic segmentation tool. Experienced medical technicians used 3D slicer open-source software [36] for further detailed refinement of spine labeling. Finally, 1012 chest CT 3D images and 40,5534 2D segmentation images of the spine were obtained. To meet the limited hardware capacity, the pixel space of CT raw data was downsampled from 0.6777 mm ± 0.0723 (mean ± SD) to 1.2766 mm ± 0.1180 (mean ± SD).

### 2.2. Generation of Simulated X-ray

**Figure 1:**
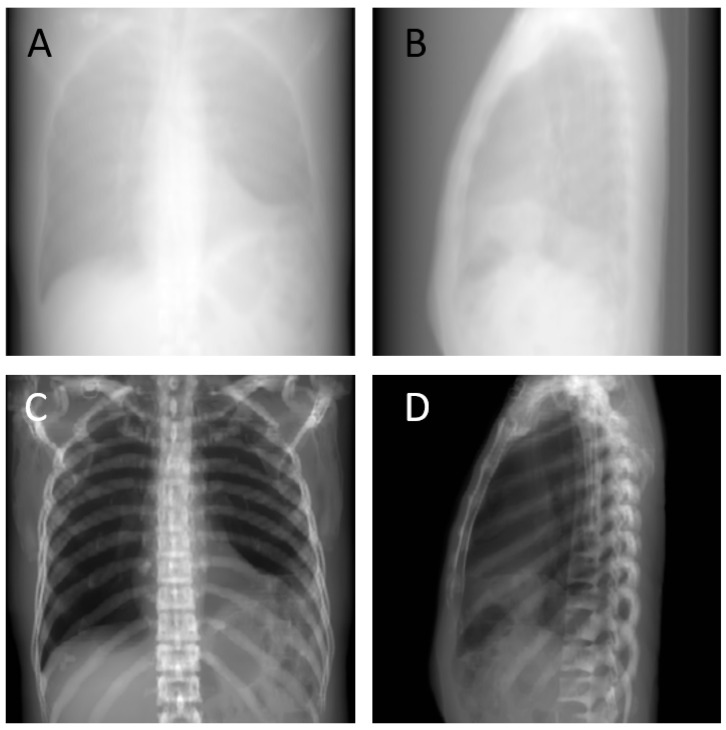
(**A**) Original simulated X-ray of AP view (**B**) Original simulated X-ray of Lat view (**C**) Enhanced bone signal simulated X-ray of AP view (**D**) Enhanced bone signal simulated X-ray of Lat view.

It is impractical and unethical to collect sufficiently synchronized original X-ray and CT images simultaneously for ML purposes. We used the previously proposed tomographic iterative GPU-based Reconstruction (TIGRE) toolbox [37] to simulate bi-planar chest X-ray images of AP and Lat views, by back-projection from cone beam CT (CBCT). The original simulated X-ray images by the TIGRE toolbox were engineering algorithm considerations, and the original simulated X-ray images manifested with a blurred appearance (Figure 1A,B). The original simulated X-ray images did not resemble real X-ray images. From a clinical radiology perspective, empirical image contrast tuning is an essential step for radiologists to evaluate the region of interest (ROI) of the images. As a result, we propose an empirical algorithm (Table 3) for enhanced bone signals on raw CT data to improve X-ray image quality. Enhanced bone signal simulated X-ray images were produced (Figure 1C,D) to approach the real X-ray images.

**Figure 2:**
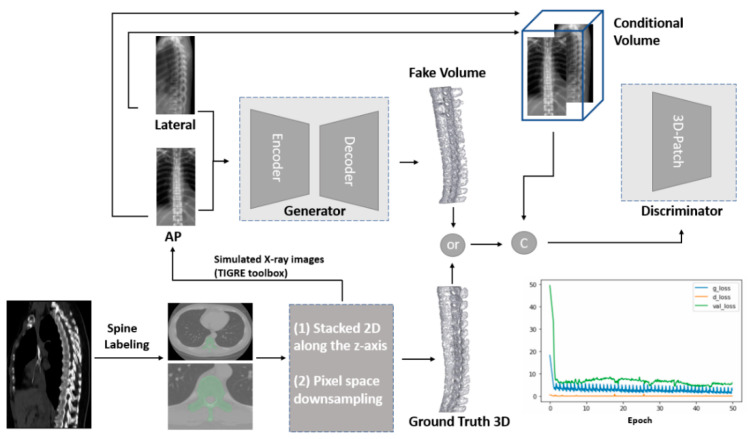
Overview of the whole architecture of the spine 2Dto3D-GAN. Circle C: Concatenate. The GT 3D spine and simulated bi-planar X-ray images were derived from CT via manual segmentation by 3D slicer software and TIGRE toolbox, respectively. The GAN model transformed 2D images into a 3D object.

The bi-planar chest X-ray images of the AP and Lat views were further cropped to bi-planar spine X-ray images with a 128 × 256 pixel size for conditional inputs into the GAN (Figure 2).

### 2.3. GAN Modeling

Our 2Dto3D-GAN of the spine (Figure 2) used simulated bi-planar spine X-ray images as conditional inputs to the generator and discriminator networks. The generator network is a modification of the X2CT-GAN [32] and discriminator network, referred to as the Patch-GAN discriminator [31]. The AP and Lat simulated X-ray images composed the conditional volume in this architecture. Therefore, this model could perform customized optimization output according to different 2D input conditions.

#### 2.3.1. Generator Design

**Figure 3:**
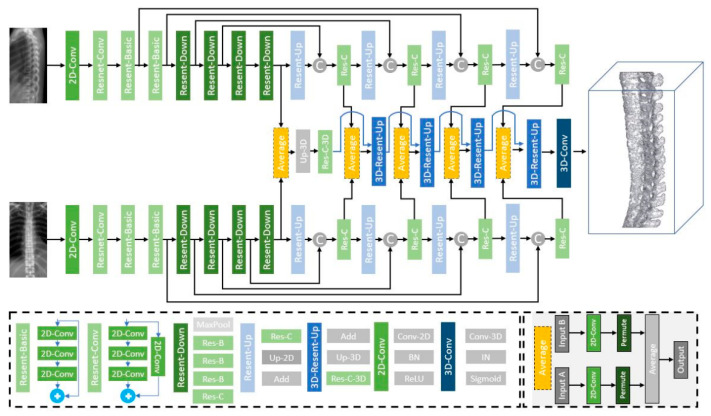
Generator network. The generator consisted of an encoder and a decoder (modified from X2CT-GAN generator [32]). Circle C: Concatenate; circle +: Add.

Our generator (Figure 3) focused on modifying the feature extraction CNN with ResNet [33] to adapt to the image dimensions and memory capacity of the hardware equipment. The original X2CT-GAN model was based on DenseNet [38] for feature extraction under spatial-domain convolution. The concept of DenseNet is similar to that of ResNet—a deep learning (DL) feature extraction architecture in the spatial domain. Furthermore, DenseNet has the concept of multilayer feature connection with higher performance than that of ResNet, but requires higher GPU memory and training time [39].

In our study, engineers and radiologists, to determine the clinically desired boundary conditions, screened the raw data and input signals. Thus, we assumed that the basic skip connection architecture of ResNet embedded in our generator could fulfill the entire training process with the limited hardware equipment.

The encoder comprised eight layers of 2D convolutional (Conv2D) layers. The first four blocks were used to extract the features, and the last four blocks were used to produce high-level features for the decoder via a skip connection. The kernel size of the first convolution layer was 7 × 7 to expand the receptive field, whereas the others had a kernel of 4 × 4 blocks. The channel sequences were (64, 64, 64, 64, 32, 16, 8, 4). The channel size was equal to the first or second dimension of the reconstruction volume, which depended on the AP or Lat view. Maximum pooling, batch normalization (BN), and ReLU activation functions were implanted between each Conv2D layer. The decoder was divided into two parts. First, the output of the encoder of the AP and Lat views was upsampling. Second, the central decoder averaged the feature maps of the AP and Lat views to reconstruct the spinal volume using a 3D convolution layer. Despite the dimensions between the AP and Lat views being different, the dimension permuted before the combination. At the beginning of the central decoder, the features were transferred to a 4 × 4 × 8 × 4 shape as the decoder input. The upper and lower decoders included four two-dimensional upsampling layers, and the central decoder consisted of five three-dimensional upsampling layers and one 3D convolution layer to increase the detail of the reconstructed volume.

The ReLU activation function was replaced by a sigmoid activation function in the final 3D convolution layer. Spine volume was only used for identification of the structural location, and we set 0.4 as a threshold to transfer output probability map to binary images with values are either “0” or “1”. Finally, a 128 × 128 × 256 3D architecture was produced in the output of the generator, as shown in Figure 3.

#### 2.3.2. Discriminator Design

**Figure 4:**
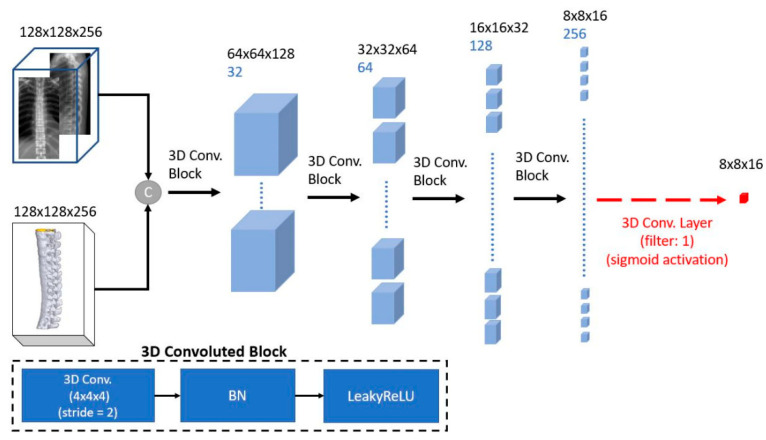
Three-dimensional-patch-GAN Discriminator. Circle C: Concatenate. The input combined conditional volume (orthogonal X-ray images) and generated 3D spine volume. Then, it was sent to the 3D Patch-GAN with four 3D convolution blocks for loss calculation.

We refer to the previously proposed Patch-GAN discriminator [31] as the 3D-Patch-GAN discriminator (Figure 4). Patch-GANs have good generalization properties and are frequently used [27,32,40,41]. Our discriminator network consisted of four 3D convolution layers (Conv3D) linked by a LeakyReLU activation layer and a BN layer. The channel sequences of the convolution layer were (32, 64, 128, 256), the kernel size was 4 × 4 × 4, and they were connected to a Conv3D at the end with a channel of 1 for the loss function calculation.

#### 2.3.3. Loss Function

The least-squares generative adversarial network (LSGAN) [42] uses different distance measurements to construct a more stable and faster convergence method for GAN and to generate more realistic images. The basic LSGAN can be divided into the discriminator LSGAN (Equation (1)) and generator LSGAN (Equation (2)).

(1) $$\underset{D}{\min}\;LSGAN\left(D\right)=\frac{1}{2}E_{x\sim p_{data\left(x\right)}}[{\left(D\left(x\right)-1\right)}^{2}]+\frac{1}{2}E_{z\sim p_{z\left(z\right)}}[{\left(D\left(G\left(z\right)\right)-0\right)}^{2}]$$

(2) $$\underset{G}{\min}\;LSGAN\left(G\right)=\frac{1}{2}E_{z\sim p_{z\left(z\right)}}\left[\left(D\left(G(z\right)\right)-1{)}^{2}\right]$$

where *G(z)* is the generation sample, *x* is the true sample, and *z* is the biplanar simulated X-ray. To increase the 3D convergence and accuracy [32], in addition to the *LSGAN*, the reconstruction loss [43] was also calculated. The reconstruction loss was used to measure the mean square error (MSE) (L2 loss) of the real and generated samples (Equation (3)).

(3) $$L_{re}=E_{z\sim p_{z}}{\left(y-G\left(z\right)\right)}^{2}$$

The final loss function is given by:

(4) $$G^{*}=\lambda_{1}\arg\min\;L_{LSGAN}\left(G,D\right)+\lambda_{2}L_{re}$$

Here $\lambda_{1}$ and $\lambda_{2}$ are set as 2, and 100, respectively.

The training process was stopped around the 50th Epoch (Figure 2).

### 2.4. Similarity Evaluation

In this study, we applied the dice similarity coefficient (*DSC*) (Equation (5)), the Jaccard similarity coefficient (*JSC*) (Equation (6)), and overlap volume (*OV*) (Equation (7)) [44,45] for volumetric comparison of GT 3D structures and GAN-predicted 3D structures.

(5) $$DSC\left(A,B\right)=\frac{2\left|A\cap B\right|}{\left|A\right|+\left|B\right|}$$

(6) $$JSC\left(A,B\right)=\frac{A\cap B}{A\cup B}$$

(7) $$OV\left(A,B\right)=\frac{A\cap B}{min\left(A,B\right)}$$

In addition to comparing 3D spatial structures, we used the structural similarity index measure (SSIM) (Equation (8)) to evaluate the perceived quality of orthogonal 2D projection images arising from GT 3D structures and GAN-predicted 3D structures.

*SSIM* [46] is a well-known objective method for evaluating the perceptual similarity between two images by combining the loss of luminance distortion (l(x,y)), contrast distortion (c(x,y)), and structural distortion (s(x,y)) (Equation (8)).

(8) SSIM(x,y)=f[l(x,y), c(x,y),s(x,y)]

In this study, we used SSIM_AP and SSIM_Lat to compare the orthogonal 2D projection from the GT spine and the predicted spine using a sliding window size of 11 × 11 pixels.

Finally, we obtained five metrics for the quantitative evaluation: *DSC*, *JSC*, *OV*, SSIM_AP, and SSIM_Lat.

### 2.5. Training Domains and Statistics

Sufficient medical data collection is not easy, and medical data labeling is laborious, which requires domain knowledge of specific medical imaging to meet the clinical environment.

**Figure 5:**
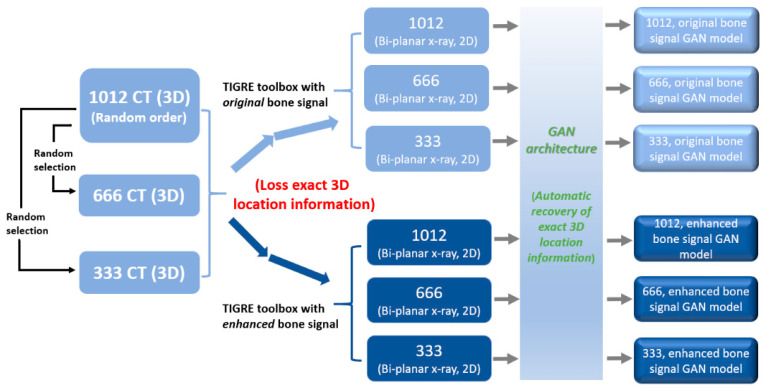
Training domains. A total of 1012 CT studies were collected. To compare the training effects with different dataset sizes, 333 and 666 CT studies were randomly selected from the dataset. Further original and enhanced bone signal conditions were processed into GAN architecture.

In this study, we designed 2 × 3 input data conditions (two different bone signal conditions × three dataset sizes) (Figure 5). We randomly selected 666 CT and 333 CT series from randomly sorted 1012 CT and further processed bone signal tuning and TIGRE toolbox transformation. The purpose of this design was to observe the effectiveness of the same DL architecture under different training portfolios.

**Figure 6:**
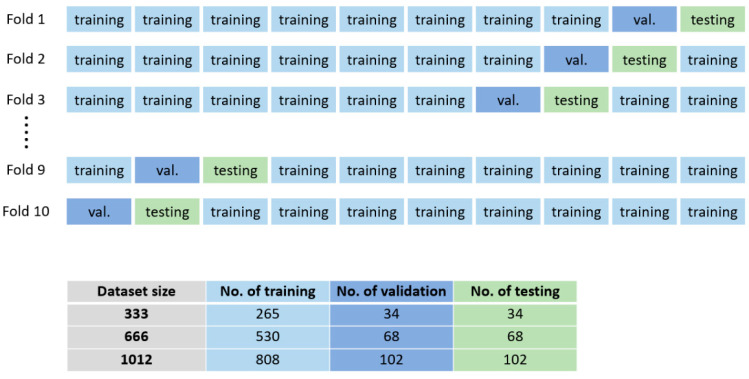
Ten-fold cross-validation. For each dataset, the training, validation, and testing ratios are 8:1:1 (numbers in table). Ten-fold cross-validation was applied to different dataset sizes.

In the 10-fold cross-validation, the ratio of training, validation, and testing was 8:1:1 for each dataset size (Figure 6). The numbers of training, validation, and testing were 808, 102, and 102 in the 1012 dataset, whereas the other numbers were 256, 34, and 34 in the 333 dataset and 530, 68, and 68 in the 666 dataset.

The Mann–Whitney U test was used to compare the training performance between the original and enhanced bone signal conditions. The Kruskal–Wallis test was used to evaluate the training performance of the three datasets. The statistical significance of the alpha value was set at 0.05.

### 2.6. Hardware and Software Equipment

This study used an NVIDIA 3090 GPU (24 GB memory size) for the GAN modeling process and an NVIDIA 2080 Ti GPU (11 GB memory size) for the TIGRE toolbox processing. The software environment included TensorFlow 2.4, Python 3.8.4, and MATLAB 2019 B on a Windows 10 operating system.

## 3. Results

### 3.1. Generation of the 3D Modeling

**Figure 7:**
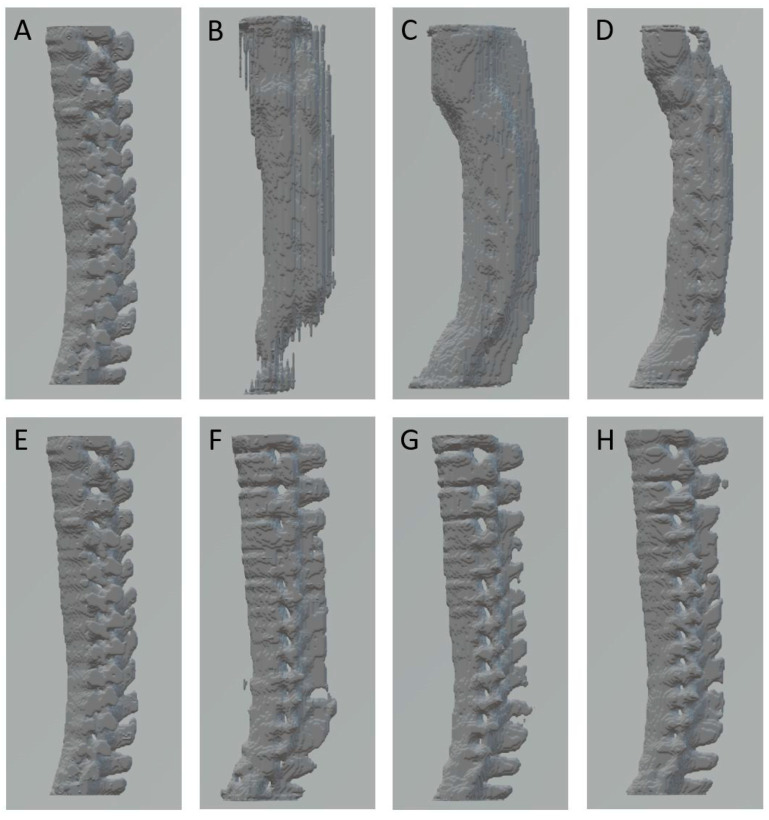
(**A**,**E**) GT spine; (**B**) Ori_333, DSC: 0.6605, SSIM_AP: 0.8293, SSIM_Lat: 0.8329; (**C**) Ori_666, DSC: 0.6620, SSIM_AP: 0.8915, SSIM_Lat: 0.8089; (**D**) Ori_1012, DSC: 0.6065, SSIM_AP: 0.8693, SSIM_Lat: 0.7880; (**F**) Enh_333, DSC: 0.8538, SSIM_AP: 0.9540, SSIM_Lat: 0.9248; (**G**) Enh_666, DSC: 0.8822, SSIM_AP: 0.9495, SSIM_Lat: 0.9417; (**H**) Enh_1012, DSC: 0.9095, SSIM_AP: 0.9649, SSIM_Lat: 0.9591. GT: ground truth; Ori_333: Original bone signals with 333 dataset size; Ori_666: Original bone signals with 666 dataset size; Ori_1012: Original bone signals with 1012 dataset size; Enh_333: Enhanced bone signals with 333 dataset size; Enh_666: Enhanced bone signals with 666 dataset size; Enh_1012: Enhanced bone signals with 1012 dataset size.

In this study, the training model stop point was set at the 50th epoch. Generator, discriminator, and validation losses were observed (Figure 2). At the training end, the trained generator network was used to produce reconstructed 3D images for human visual perception and quantitative analysis. After completing the model training, we tested the timing from the input of bi-planar data to the automatic production of 3D volume data for one case, which was 0.306 s under an Nvidia RTX 3090 GPU and 1.725 s under an Intel I5-8400 CPU. This case is shown in Figure 7 and supplementary material in Video S1: 3D spine rotation.mp4. Figure 7A,E demonstrate the GT for the same case.

The predicted 3D spines under the original bone signal conditions in the 333, 666, and 1012 dataset sizes are depicted in Figure 7B–D. For bone-enhanced signal conditions in the 333, 666, and 1012 dataset sizes, the predicted 3D spine images are depicted in Figure 7F–H, respectively. We subjectively observed that the contours of the predicted 3D spine under enhanced bone signal conditions (Figure 7F–H) were better than those under the original bone signal conditions (Figure 7B–D). As the dataset size increased under the original bone signal condition, we observed that the predicted structure was an irregular contour of a column (Figure 7B), further evolution of the gross structures with vertebral bodies and spinous process (Figure 7C), and grossly segmented discrimination of different vertebrae (Figure 7D) was observed in the larger training size groups.

In the enhanced bone signal condition, ambiguous segmental discrimination of different vertebrae and sticky spinous processes were observed in the dataset size 333 (Figure 7F). The enhanced bone signal models from the 666 and 1012 datasets produced similar predicted 3D spines (Figure 7G,H).

### 3.2. Quantitative Performance Assessment

**Figure 8:**
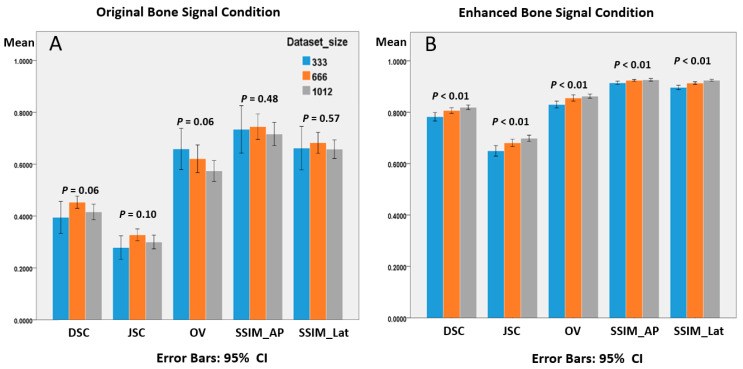
(**A**,**B**) Training performance between different training dataset sizes in original and enhanced bone signal conditions. CI: confidence interval.

The quantitative results for the six training conditions are presented in Table 4 and Figure 8. These metrics were evaluated in two dimensions: different bone signal conditions and different dataset sizes.

Considering the different bone signal conditions, there were significant † *p*-values (<0.01) of all metrics for comparison of the original and enhanced bone signal conditions regardless of different dataset sizes (last column in Table 4). The DSC values under original signal conditions were between 0.4 and 0.45, and approximately 0.8 under enhanced bone signal conditions. The optimal mean DSC value for all conditions was 0.8192, which was in the enhanced-1012 dataset size group, and the training performance was approximately twice that of the original signal conditions (0.8192 vs. 0.4177). The JSC values under the original signal condition were between 0.28 and 0.33, and between 0.65 and 0.70 under the enhanced signal condition. The optimal mean JSC value for all conditions was 0.6984, which was in the enhanced 1012 training group, and the training performance ratio was 2.32 (0.6984 vs. 0.3012). The OV values under original signal conditions were between 0.57 and 0.66, and approximately 0.85 under enhanced bone signal conditions. The optimal mean OV value for all conditions was 0.8624, which was in the enhanced 1012 training group, and the training performance ratio was 1.51 (0.8624 vs. 0.5730).

The SSIM_AP values in the original signal conditions ranged from 0.7 to 0.74 and were approximately 0.92 under enhanced bone signal conditions. The SSIM_Lat values under the original signal conditions ranged from 0.65 to 0.68 and were approximately 0.91 under enhanced bone signal conditions.

All DSC and SSIM values demonstrated statistically significant performance improvement after empirical bone enhancement.

Considering the increasing dataset size of the training processes, we could observe that the larger the training dataset size, the better the evaluation values, with statistical significance under enhanced bone signal conditions (Figure 8B). However, under the original bone signal conditions, the training effect did not increase correspondingly (Figure 8A). In addition, the range of error bars of 10-fold cross-validation under the original bone signal condition was larger than that under the enhanced bone signal condition (Figure 8A,B).

## 4. Discussion

The DL technique has received extensive attention in recent years, providing an optimal solution method for more complicated conditions. The central idea of this study is purposing a cost-effective auxiliary diagnostic method to current diagnostic imaging modalities rather than to develop a gold standard functional diagnostic tool with advanced capability.

In this study, we modified the X2CT-GAN to 2Dto3D-GAN of the spine to demonstrate the feasibility of the automatic transformation of 2D bi-planar spine images to 3D structures. This research followed the development over the past 40 years in the fields of biomedical and computer vision studies.

The engineering characteristics of 2Dto3D-GAN of the spine are as follows: (1) GAN is a combination of two neural networks with a generator and a discriminator. This can provide additional optimization trade-off points according to the input dataset. (2) The conditional volume in Figure 2 provides the training constraint for the GAN architecture. It allows this neural network to adapt to different biplanar AP and Lat images with their own acquisition parameters and thickness of the 2D structures. (3) The 3D patch discriminator can provide a more detailed evaluation of each component (patch) of the entire structure. These characteristics of 2Dto3D-GAN can provide a more generalized DL architecture design (generalization ability) for diverse input datasets.

The radiology characteristic of 2Dto3D-GAN of the spine is that in most conditions, medical images have only one channel of grayscale values, under diverse anatomic and pathophysiological imaging features, and structures that are different from the engineering application situation. Although CNN has been a well-known method in recent years for feature extraction and generalization, we introduced a clinical-perspective empirical method to adjust the input signals to approach real situations. Our study showed that appropriate regulation of input signals is an important initial step for individual-specific DL purposes under domain knowledge.

This study conducted random sorting and selection for raw data. However, in a standard medical cohort study, training (including validation) and testing datasets should be processed at different centers. In fact, the environmental conditions of medical engineering in different hospitals are different, and patient physical characteristics are diverse, which in turn affects the original signal variations of the same type of imaging modality. According to our limited experience, the design, DL architecture, and training portfolios should meet the clinical requirements. Most DL projects are often unable to simultaneously build a perfect model at one time. It needs to start with a basic model through transfer learning and adjust detailed parameters to meet the final clinical needs. Thus, building a reliable prototype that mimics the final medical requirements is an essential step in DL model development. This architecture can provide the essential basic parameters for multicenter conduction in the future.

One of the vulnerable defects of our current model is that it is a basic prototype model with constrained training portfolios rather than training on generalized actual medical situations and conducted in multiple centers. Another defect is that the 2D simulated X-ray images were derived from GT 3D structures and lost some exact 3D location information (Figure 5). We did not evaluate transformation errors from the internal system of the TIGRE toolbox for the similarity of real X-ray images and simulated X-ray images.

As we mentioned in the introduction, the purposes of this architecture are to improve the semi-automatic workflow of the EOS system and provide auxiliary diagnostic information on standard X-ray images. Therefore, the training portfolio of our study was similar to that of the EOS system with bi-planar X-ray image inputs and 3D image output. If permitted by the EOS system, we could use EOS bi-planar images and EOS semi-automatic GT 3D output in our current GAN model to train and validate an automatic 2D/3D registration workflow. For application to real bi-planar-X-ray images, we must solve the requirement of the spatial alignment of AP and Lat X-ray images and signal adjustment as input data pairs to our current model. These required more testing for the final clinical demand and environment of imaging modalities in future research.

For evaluation of training performance in volumetric metrics, the Dice value was around 0.8, JSC value was around 0.68, and OV value was around 0.85 in enhanced bone signals (Table 4). We observed a gradual increment in training performance (Figure 8B). We also noted indistinct disc spaces, blunting borders of transverse processes, and blunting border of spinous processes in GT 3D and predicted 3D structures in the enhanced condition (Figure 7F–H). This could be further elucidated from three perspectives. First, to meet the limited hardware capacity, a downsampling interpolation process for the CT raw data was performed in this experiment. This led to a natural deficiency in feature extraction. Upsampling of raw data can be tested in our future work to produce more delicate 3D structures. Second, there are two major domains for digital imaging processing: spatial domain analysis in Euclidean space and frequency domain by Fourier transform. This prototype architecture was based on a spatial domain and applied the basic feature extraction block of ResNet. Third, this architecture focused on the structure information (0 or 1) without further inferring the density spectrum of each pixel (0–255 values), owing to hardware limitations. This could lead to an indistinct border in predicted 3D structures. In a follow-up task, a more complicated spatial domain feature extraction network or mixture frequency domain features can be studied for real X-ray images, and this can be conducted in multiple centers using transfer learning techniques to satisfy appropriate clinical requirements.

In this study, we proved the theoretical possibility of automatic 2D/3D registration of the spine using a GAN model and further elucidated the essential implementation details of this architecture. Our study demonstrates the potential clinical application of the DL technique of GAN to adapt to human structural diversities in automatic trends and provide more auxiliary diagnostic information to current imaging modalities.

## 5. Conclusions

This study implemented a GAN model for automatic different-dimensional image transformation of the spine with the potential for clinical application in a routine examination. It is hoped that a better DL model and multicenter conduction can be achieved with low-cost, faster, high-quality, and accurate 3D spine reconstruction from a novel perspective.

## Figures and Tables

**Table 1:** Demographic distribution of the study population.

Characteristic		Number	Percentage (%)
**Sex**	Female	159	16.16
	Male	217	22.05
	N/A	608	61.79
**Age group (y)**	19–29	17	1.73
	30–39	37	3.76
	40–49	121	12.30
	50–59	163	16.57
	60–69	21	2.13
	70–84	17	1.73
	N/A	608	61.78
	Total	984	-

N/A: not available.

**Table 2:** Parameters of CT.

	Characteristic	No. of CT	Percentage (%)
**Manufacture**	General Electric	582	57.51
	Siemens	426	42.09
	N/A	4	0.4
**Scan Options**	Helical Mode	583	57.61
	N/A	429	42.39
**Slice Thickness (mm)**	0.6–3	466	46.05
	5	543	53.66
	10	3	0.29
**Tube Voltage (kVp)**	100	7	0.69
	120	760	75.10
	130	168	16.60
	140	77	7.61
**Tube Current (mA)**	0–100	401	39.62
	101–200	271	26.78
	201–300	144	14.23
	301–400	89	8.80
	401–500	43	4.25
	501–1100	64	6.32
**Reconstruction Diameter/** **Field of view (FOV) (mm)**	200–300	118	11.66
	301–350	416	41.11
	351–400	413	40.81
	401–500	65	6.42

N/A: not available.

**Table 3:** Algorithm of enhanced bone signals.

Steps	
**(1)**	Read CT image values from a 3D slicer in Hounsfield unit (HU).
**(1.1)**	Set the low HU value threshold filter as: if image value <−1024, then image value = −1024.
**(1.2)**	Set the high HU value threshold filter as: if image value >1000, image value = 0.
**(1.3)**	Set the empirical mask filter: if image value >−400, mask pixel value = 1 else, mask pixel value = 0.
**(1.4)**	Apply mask filter: mask filter (1.3) × [image values filtered by (1.1) (1.2)].
**(2)**	Convert filtered image values into imaging signal values. Imaging signal value = (filtered image value-DicomInfo.RescaleIntercept)\DicomInfo.RescaleSlope
**(2.1)**	Set the empirical specific windowing as: if imaging signal value ≤874 or ≥2024, then imaging signal value = 0.
**(2.2)**	A higher imaging signal value was enhanced. increase imaging signal value three times if imaging signal value >1300.

**Table 4:** Evaluation metrics of different training conditions.

Evaluation Metrics	Bone Signal Conditions	333 Dataset Mean (SD)	666 Dataset Mean (SD)	1012 Dataset Mean (SD)	* *p*-Value	† *p*-Value
**DSC**	**Original**	0.3950 (0.0863)	0.4533 (0.0329)	0.4177 (0.0421)	0.06	<0.01
**JSC**		0.2788 (0.0635)	0.3276 (0.0318)	0.3012 (0.0370)	0.10	
**OV**		0.6587 (0.1112)	0.6211 (0.0744)	0.5730 (0.0557)	0.06	
**SSIM_AP**		0.7342 (0.1280)	0.7446 (0.0684)	0.6992 (0.0626)	0.48	
**SSIM_Lat**		0.6619 (0.1168)	0.6825 (0.0567)	0.6464 (0.0501)	0.57	
**DSC**	**Enhanced**	0.7825 (0.0229)	0.8068 (0.0152)	0.8192 (0.0121)	<0.01	
**JSC**		0.6497 (0.0284)	0.6807 (0.0198)	0.6984 (0.0170)	<0.01	
**OV**		0.8300 (0.0184)	0.8553 (0.0172)	0.8624 (0.0112)	<0.01	
**SSIM_AP**		0.9143 (0.0090)	0.9238 (0.0049)	0.9261 (0.0073)	<0.01	
**SSIM_Lat**		0.8966 (0.0116)	0.9135 (0.0070)	0.9242 (0.0054)	<0.01	

DSC: dice similarity coefficient; JSC: Jaccard similarity coefficient; OV: overlap volume; SSIM: structural similarity index measure; AP: anteroposterior; Lat: lateral; SD: standard deviation; * *p*-value: Kruskal–Wallis test; † *p*-value: Mann–Whitney U test.

## Data Availability

Not applicable.

## Supplementary Materials

The following supporting information can be downloaded at: https://www.mdpi.com/article/10.3390/diagnostics12051121/s1, Video S1: 3D spine rotation.mp4.
